# Efficacy of Losartan Potassium and Benazepril in Hypertensive Patients With Insulin Resistance: Impact on Blood Pressure, Insulin Sensitivity, and Diabetes Risk

**DOI:** 10.7759/cureus.80833

**Published:** 2025-03-19

**Authors:** Ahmed Chaudhary, Sehar Sehar, Sana Iqbal, Muhammad Noor, Ayesha Faisal, Saddam Hussain, Marym Khan, Khaqan Ahmed, Afsheen Siddiqi, Talha Mazhar

**Affiliations:** 1 Internal Medicine, Michigan State University, Detroit, USA; 2 Internal Medicine, Lahore Medical and Dental College, Lahore, PAK; 3 Internal Medicine, Dow University of Health Sciences, Civil Hospital Karachi, Karachi, PAK; 4 Internal Medicine, DMC Sinai-Grace Hospital, Detroit, USA; 5 Geriatrics, Liverpool University Hospitals NHS Foundation Trust, Liverpool, GBR; 6 Medicine, CMH Kharian Medical College, Kharian, PAK; 7 Diabetes and Endocrinology, University Hospital Coventry and Warwickshire, Coventry, GBR; 8 General Internal Medicine, Khyber Teaching Hospital, Peshawar, PAK; 9 Medicine, Shifa International Hospitals, Islamabad, PAK; 10 Medicine, Khyber Teaching Hospital, Peshawar, PAK; 11 Pharmacology and Therapeutics, Ayub Medical College, Abbottabad, PAK; 12 Medicine and Surgery, Saidu Medical College, Swat, PAK; 13 Medicine and Surgery, Divisional Headquarters Teaching Hospital, Mirpur, PAK

**Keywords:** benazepril, blood pressure, homa-ir, hypertension, insulin resistance, losartan potassium

## Abstract

Background

Hypertension and insulin resistance (IR) often coexist, significantly increasing the risk of type 2 diabetes and cardiovascular disease. IR plays a key role in metabolic syndrome, promoting endothelial dysfunction and impairing glucose metabolism. Angiotensin receptor blockers (ARBs) and angiotensin-converting enzyme inhibitors (ACEIs) are commonly prescribed for hypertension, but their differential effects on insulin sensitivity and diabetes risk remain unclear. This study compares the effects of losartan potassium (LP) (ARBs) and benazepril (ACEIs) on blood pressure control, insulin sensitivity, and metabolic parameters in hypertensive patients with IR.

Methods

A prospective cohort study was conducted from September 2023 to September 2024 at Khyber Teaching Hospital, Peshawar and Lahore Medical and Dental College, Lahore. A total of 364 hypertensive patients with IR were included. Based on physician-prescribed treatment, patients were categorized into two groups: Group A received benazepril (10 mg/day), and Group B received LP (50 mg/day). Primary outcomes included blood pressure control, Homeostatic Model Assessment for Insulin Resistance (HOMA-IR) scores, fasting glucose, and lipid profiles, measured at baseline and regular follow-ups over 12 months. Patient adherence was monitored through self-reports and prescription refill checks. Missing data were handled using multiple imputations. Statistical analyses included independent t-tests, paired t-tests, repeated-measures ANOVA, and multivariate regression models, with a significance level set at p < 0.05.

Results

Both treatments significantly reduced blood pressure, but LP demonstrated greater efficacy, with superior reductions in systolic (26.4 mmHg vs. 24.7 mmHg) and diastolic (15.3 mmHg vs. 13.6 mmHg) readings (p < 0.05). HOMA-IR scores improved more in the losartan group (1.7 vs. 1.4; p = 0.02), along with a greater decrease in fasting glucose, suggesting a potential protective effect against diabetes development. Lipid profile improvements were comparable between groups. Adherence rates were similar, with a dropout rate of 8.2% and no major adverse events.

Conclusions

LP was more effective than benazepril in lowering blood pressure and improving insulin sensitivity, which may contribute to a lower diabetes risk in hypertensive patients with IR. A randomized controlled trial with longer follow-up is recommended to confirm these findings and assess long-term metabolic outcomes.

## Introduction

One of the biggest threats to world health is hypertension, which also increases the risk of cardiovascular disease and death. A key component of metabolic syndrome, it frequently coexists with other metabolic conditions, such as insulin resistance (IR) [1]. Approximately 50-60% of hypertensive patients exhibit IR, significantly increasing their risk of cardiovascular events and metabolic complications. Through processes including elevated sympathetic activity, compromised endothelial function, and activation of the renin-angiotensin-aldosterone system, IR - which is defined by the decreased cellular absorption of glucose in response to insulin - exacerbates hypertension. Elevated sympathetic activity not only increases blood pressure via vasoconstriction and sodium retention but also worsens IR by impairing glucose uptake and increasing hepatic glucose production. Therefore, an integrated strategy that targets both blood pressure and metabolic abnormalities is necessary for the efficient therapy of hypertensive patients with IR [2,3].

Renin-angiotensin system (RAS) inhibitors, particularly angiotensin receptor blockers (ARBs) and angiotensin-converting enzyme inhibitors (ACEIs), play a crucial role in hypertension management [4]. Beyond their primary function of lowering blood pressure, these agents also modulate insulin sensitivity and improve metabolic outcomes. Losartan potassium (LP), an ARB, selectively blocks the angiotensin II type 1 receptor, thereby reducing vasoconstriction, inflammation, and oxidative stress factors known to impair insulin sensitivity. Additionally, it has been shown to activate peroxisome proliferator-activated receptor gamma (PPAR-γ), which enhances glucose uptake and lipid metabolism [5,6]. In contrast, benazepril, an ACEI, inhibits the conversion of angiotensin I to angiotensin II, leading to vasodilation and improved endothelial function. However, the metabolic impact of benazepril remains less well defined, with some studies suggesting a neutral or mild beneficial effect on IR, primarily due to its anti-inflammatory and vascular benefits [7,8].

While randomized controlled trials and mechanistic studies have demonstrated losartan’s favorable effects on insulin sensitivity, fewer studies have explored benazepril’s metabolic effects in detail. Moreover, despite extensive research on ARBs and ACEIs individually, there is no direct head-to-head comparison of losartan and benazepril in hypertensive patients with IR [9]. Indirect evidence from meta-analyses suggests ARBs may offer superior metabolic benefits compared to ACEIs in patients with metabolic syndrome, but specific comparative data remain lacking [10,11]. Understanding these differences is essential for optimizing treatment strategies in this high-risk population.

Objectives

This prospective cohort study evaluates the real-world effectiveness of LP and benazepril in managing hypertension among insulin-resistant patients at Khyber Teaching Hospital (KTH), Peshawar and Lahore Medical and Dental College (LMDC), Lahore, over 12 months (October 2023 to September 2024). Patients receiving either medication as part of routine clinical care were observed for changes in blood pressure, IR (where assessed), fasting glucose, lipid profile, and adverse events. Treatment decisions were made independently by physicians, following established guidelines, without researcher intervention. The study aims to compare treatment outcomes, identify factors influencing response, and ensure methodological rigor through appropriate statistical adjustments and standardized data collection, maintaining its observational nature.

## Materials and methods

Ethical statement

The study was approved by the Institutional Research and Ethical Review Board of Khyber Medical College/KTH, Peshawar, on August 17, 2023. Written informed consent was obtained from all participants before enrollment, including consent for treatment and open-access publication of findings. Confidentiality was strictly maintained, and participants had the right to withdraw from the study at any time, without affecting their routine medical care. The study adhered to the principles of the Declaration of Helsinki. There were no financial disclosures, conflicts of interest, or external funding sources declared.

Study design and setting

This prospective observational cohort study was conducted at KTH, Peshawar, and LMDC, Lahore, from September 2023 to September 2024. The study aimed to evaluate the real-world comparative effectiveness of LP and benazepril in managing hypertension among patients with IR and increased diabetes risk.

Treatment decisions were entirely at the discretion of physicians, following standard clinical practice and established guidelines, including the Joint National Committee (JNC 8) and the American Diabetes Association (ADA) recommendations for hypertension management in insulin-resistant individuals. The study did not assign medications, randomize patients, or intervene in prescribing practices, ensuring that the results reflect real-world clinical management.

Sample size calculation

The sample size was determined based on the global prevalence of IR among hypertensive patients, estimated at 27% in a representative cohort study [12]. Using a standard formula for comparative studies, with a 95% confidence level, 80% statistical power, and an anticipated 10% dropout rate, the required sample size was 182 participants per group, totaling 364 patients. This prevalence aligns with global epidemiological data, considering the substantial burden of hypertension-related cardiovascular mortality, which accounts for over seven million deaths annually, as reported by WHO [12].

Study population and sampling

Participants were prospectively recruited from outpatient departments at KTH and LMDC and followed for 12 months to assess changes in blood pressure, insulin sensitivity, and metabolic parameters. The study included patients aged 30-70 years with a confirmed diagnosis of hypertension and IR, defined as a Homeostatic Model Assessment for Insulin Resistance (HOMA-IR) score of ≥2.5, a threshold validated in previous research. To ensure homogeneity, patients with secondary hypertension, significant renal or hepatic impairment, or those receiving other insulin-sensitizing therapies such as metformin, sodium-glucose cotransporter-2 (SGLT2) inhibitors, or glucagon-like peptide-1 (GLP-1) receptor agonists were excluded.

Treatment allocation and adherence monitoring

Patients were assigned to one of two exposure groups based on routine clinical decisions made by their physicians. The first group comprised patients prescribed benazepril (10 mg/day), while the second group included those prescribed LP (50 mg/day). Since this was an observational study, randomization and blinding were not performed. However, selection bias was minimized by adhering to strict inclusion criteria and adjusting for confounding variables during statistical analysis.

Medication adherence was classified into three categories: high adherence (≥90%), moderate adherence (70-89%), and low adherence (<70%). Adherence monitoring was conducted through patient self-reports, pill counts during monthly follow-ups, and pharmacy refill records when available. Participants were considered adherent if they had taken at least 90% of their prescribed doses throughout the study period.

Data collection and follow-up

Baseline demographic and clinical data, including age, gender, BMI, blood pressure, fasting glucose levels, lipid profile, and IR (HOMA-IR), were recorded (Table 1). Follow-up assessments were conducted monthly for blood pressure measurements, adherence assessments, and adverse event monitoring. IR was reassessed every three months, while lipid profile and fasting glucose levels were evaluated every six months.

**Table 1:** Follow-up schedule precision HOMA-IR, Homeostatic Model Assessment for Insulin Resistance; IR, insulin resistance

Outcome measure	Follow-up interval
Blood pressure	Monthly
Adherence assessment	Monthly
IR (HOMA-IR)	Every three months
Lipid profile and fasting glucose	Every six months
Adverse event monitoring	At each visit

Antihypertensive treatment, including dose selection and adjustments, was determined exclusively by physicians at KTH and LMDC, following routine clinical assessments and standard guidelines such as JNC 8 and ADA recommendations. Patients were initially prescribed LP (50 mg/day) or benazepril (10 mg/day), the standard starting doses for hypertension management. Adjustments were made based on individual patient needs, including blood pressure control, tolerability, and coexisting conditions. While dose modifications - including increases, decreases, or medication switches - were recorded, they were not study-driven. Patients remained within their assigned exposure groups regardless of dose changes, and statistical analyses accounted for these variations using the intention-to-treat approach and multiple imputation techniques to handle missing data.

Medication adherence was monitored through patient self-reports, pill counts during monthly follow-ups, and pharmacy refill records (when available). Participants were classified as adherent if they had taken at least 90% of their prescribed doses throughout the study period. The adherence rates between the two treatment groups were analyzed to assess treatment consistency and its potential impact on clinical outcomes.

At each follow-up visit, participants were systematically questioned about any adverse events they experienced. Reported side effects were documented and categorized as mild, moderate, or severe based on predefined clinical criteria. The most frequently reported adverse events included dizziness and mild gastrointestinal discomfort. Incidence rates of these side effects were recorded and statistically analyzed to compare differences between treatment groups. While the study was prepared to document and report severe adverse events or treatment discontinuations due to side effects, no such cases occurred during the study period. Patients continued within their assigned exposure groups regardless of dose adjustments, with statistical analyses factoring in these variations through intention-to-treat principles and multiple imputation techniques.

Outcome measures

The primary outcome of the study was the reduction in systolic and diastolic blood pressure over the 12-month period. Secondary outcomes included changes in HOMA-IR values, fasting glucose levels, lipid profile, and the incidence of adverse events. A clinically meaningful metabolic improvement was defined as a ≥10% reduction in HOMA-IR or a ≥0.5 mmol/L decrease in fasting glucose levels.

Bias reduction strategies

Several measures were taken to minimize bias in the study. Selection bias was addressed by consecutively enrolling all eligible patients and applying strict inclusion criteria. To account for potential confounders, multivariable regression models were used to adjust for factors such as age, BMI, and baseline blood pressure. Standardized protocols were followed throughout data collection, and laboratory assessments were conducted using consistent equipment to ensure accuracy. Observer bias was reduced by ensuring that trained healthcare professionals performed blood pressure measurements using a standardized method. Missing data were managed using multiple imputation techniques and sensitivity analyses to ensure the robustness of the findings.

Statistical analysis

Statistical analysis was performed using IBM SPSS Statistics for Windows, Version 25.0 (Released 2017; IBM Corp., Armonk, NY, USA). Descriptive statistics were used to summarize baseline characteristics, with means and SDs reported for continuous variables and frequencies and percentages for categorical variables. To compare baseline differences between treatment groups, independent sample t-tests and chi-square tests were applied. Changes within groups over time were analyzed using paired t-tests and repeated-measures ANOVA, while two-way ANOVA was employed to assess interaction effects between treatment groups and subgroups, such as age and BMI. For subgroup analyses, patients were categorized based on BMI into two groups: BMI <30 kg/m² (Group A) and BMI ≥30 kg/m² (Group B).

To control for potential confounding variables, multivariate linear regression models were applied to adjust for baseline imbalances. Although propensity score matching was initially considered to improve group comparability, it was not implemented due to sample size constraints. Instead, multivariate regression models were used to adjust for key confounders, including age, BMI, and baseline metabolic parameters. Effect sizes were calculated to determine the clinical relevance of observed differences. A p-value of <0.05 was considered statistically significant, and 95% CIs were reported for key outcome measures.

## Results

A total of 182 participants were included in the study and categorized into two groups based on physician-prescribed treatment: Group A (91 participants received LP) and Group B (91 participants received benazepril). Table 2 presents the baseline characteristics of the participants. There were no significant differences between the groups in terms of age, gender distribution, BMI, baseline blood pressure, HOMA-IR scores, fasting glucose levels, lipid profiles, or serum creatinine levels (p > 0.05), suggesting comparability at baseline. Appropriate statistical adjustments were made in the analysis to account for potential confounding variables. Participants in Group A were 54.2 ± 9.8 years old on average, whereas those in Group B were 53.7 ± 10.1 years old (p = 0.72). There were 54.9% more men and 45.1% more women in Group A and 56.0% more men and 44.0% more women in Group B, respectively (p = 0.87). At baseline, there were no statistically significant differences between the groups in terms of HOMA-IR scores, fasting glucose levels, lipid profiles, or serum creatinine levels (p > 0.05).

**Table 2:** Baseline characteristics of participants DBP, diastolic blood pressure; HDL, high-density lipoprotein; HOMA-IR, Homeostatic Model Assessment for Insulin Resistance; LDL, low-density lipoprotein; SBP, systolic blood pressure

Parameter		Group A (n = 91)	Group B (n = 91)	Mean difference (95% CI)	Test statistic	p-Value	Effect size
Age (years)	Mean ± SD	54.2 ± 9.8	53.7 ± 10.1	0.5 (-2.9, 3.9)	0.35 (t)	0.72	d = 0.05
Gender	M	50	51	-	0.03 (χ²)	0.87	V = 0.008
	F	41	40				
BMI (kg/m²)	Mean ± SD	28.6 ± 2.4	28.8 ± 2.6	-0.2 (-1.1, 0.7)	0.41 (t)	0.68	d = 0.08
SBP (mmHg)	Mean ± SD	154.8 ± 10.6	155.3 ± 10.1	-0.5 (-4.2, 3.2)	0.24 (t)	0.81	d = 0.05
DBP (mmHg)	Mean ± SD	96.5 ± 5.8	97.0 ± 6.1	-0.5 (-2.9, 1.9)	0.36 (t)	0.72	d = 0.08
HOMA-IR	Mean ± SD	4.8 ± 1.2	4.9 ± 1.1	-0.1 (-0.5, 0.3)	0.28 (t)	0.78	d = 0.08
Fasting glucose (mg/dL)	Mean ± SD	132.5 ± 14.6	131.9 ± 15.0	0.6 (-5.1, 6.3)	0.19 (t)	0.84	d = 0.04
LDL cholesterol (mg/dL)	Mean ± SD	138.2 ± 10.3	137.5 ± 9.8	0.7 (-3.2, 4.6)	0.33 (t)	0.74	d = 0.07
HDL cholesterol (mg/dL)	Mean ± SD	42.8 ± 5.6	43.2 ± 5.4	-0.4 (-2.1, 1.3)	0.43 (t)	0.67	d = 0.07
Serum creatinine (mg/dL)	Mean ± SD	1.06 ± 0.12	1.05 ± 0.11	0.01 (-0.03, 0.05)	0.22 (t)	0.82	d = 0.08

Blood pressure reduction was observed in both groups over the 12-month study period, as shown in Table 3, which is placed after this paragraph. In Group A, the mean systolic blood pressure (SBP) decreased from 154.8 ± 10.6 mmHg to 128.4 ± 7.2 mmHg, while in Group B, it decreased from 155.3 ± 10.1 mmHg to 130.6 ± 7.5 mmHg. The reduction in SBP was significantly greater in Group A compared to Group B (mean reduction: 26.4 mmHg vs. 24.7 mmHg, p = 0.03). Similarly, diastolic blood pressure (DBP) decreased significantly in both groups. In Group A, the mean DBP decreased from 96.5 ± 5.8 mmHg to 81.2 ± 4.9 mmHg, whereas in Group B, it decreased from 97.0 ± 6.1 mmHg to 83.4 ± 5.1 mmHg. The difference in DBP reduction between the two groups was statistically significant (p = 0.04).

**Table 3:** Changes in blood pressure DBP, diastolic blood pressure; SBP, systolic blood pressure

Parameter	Baseline	Month 6	Month 12	F-value	p-value
SBP (Group A)	154.8 ± 10.6	137.2 ± 8.3	128.4 ± 7.2	4.85	0.03
SBP (Group B)	155.3 ± 10.1	139.0 ± 8.7	130.6 ± 7.5	4.76	0.03
DBP (Group A)	96.5 ± 5.8	87.6 ± 5.3	81.2 ± 4.9	4.19	0.04
DBP (Group B)	97.0 ± 6.1	89.2 ± 5.6	83.4 ± 5.1	4.28	0.04

Table 4 summarizes the changes in HOMA-IR scores, fasting glucose, and lipid parameters over the study period. In Group A, HOMA-IR scores significantly decreased from 4.8 ± 1.2 at baseline to 3.1 ± 1.0 at 12 months (p < 0.001, Cohen’s d = 1.42, η² = 0.05). Similarly, in Group B, HOMA-IR scores declined from 4.9 ± 1.1 to 3.5 ± 1.1 (p < 0.001, Cohen’s d = 1.15, η² = 0.05). The between-group comparison showed a greater reduction in Group A (mean difference: 1.7 vs. 1.4, p = 0.02). Fasting glucose levels significantly declined in both groups, with Group A showing a reduction from 132.5 ± 14.6 mg/dL to 113.9 ± 10.8 mg/dL (p = 0.04, Cohen’s d = 1.18, η² = 0.04) and Group B from 131.9 ± 15.0 mg/dL to 117.0 ± 11.2 mg/dL (p = 0.04, Cohen’s d = 0.97, η² = 0.04). Low-density lipoprotein (LDL) cholesterol levels exhibited within-group reductions, with Group A decreasing from 138.2 ± 10.3 mg/dL to 126.7 ± 7.6 mg/dL (p = 0.06, Cohen’s d = 0.81, η² = 0.03) and Group B from 137.5 ± 9.8 mg/dL to 129.0 ± 8.2 mg/dL (p = 0.07, Cohen’s d = 0.78, η² = 0.03). However, the between-group differences were not statistically significant (p > 0.05).

**Table 4:** Changes in HOMA-IR and metabolic parameters HOMA-IR, Homeostatic Model Assessment for Insulin Resistance; LDL, low-density lipoprotein

Parameter	Baseline	Month 3	Month 6	Month 9	Month 12	F-value	p-value	Cohen’s d	η²
HOMA-IR (A)	4.8 ± 1.2	4.3 ± 1.1	3.8 ± 1.1	3.4 ± 1.0	3.1 ± 1.0	5.23	0.02	1.42	0.05
HOMA-IR (B)	4.9 ± 1.1	4.5 ± 1.1	4.0 ± 1.1	3.7 ± 1.1	3.5 ± 1.1	5.18	0.02	1.15	0.05
Fasting glucose (A)	132.5 ± 14.6	125.3 ± 13.2	120.1 ± 12.0	116.8 ± 11.4	113.9 ± 10.8	4.02	0.04	1.18	0.04
Fasting glucose (B)	131.9 ± 15.0	127.8 ± 13.5	122.5 ± 12.4	119.4 ± 11.9	117.0 ± 11.2	4.08	0.04	0.97	0.04
LDL cholesterol (A)	138.2 ± 10.3	135.4 ± 9.6	132.1 ± 8.4	129.5 ± 8.1	126.7 ± 7.6	3.61	0.06	0.81	0.03
LDL cholesterol (B)	137.5 ± 9.8	135.9 ± 9.4	133.8 ± 9.1	131.3 ± 8.6	129.0 ± 8.2	3.42	0.07	0.78	0.03

**Figure 1:**
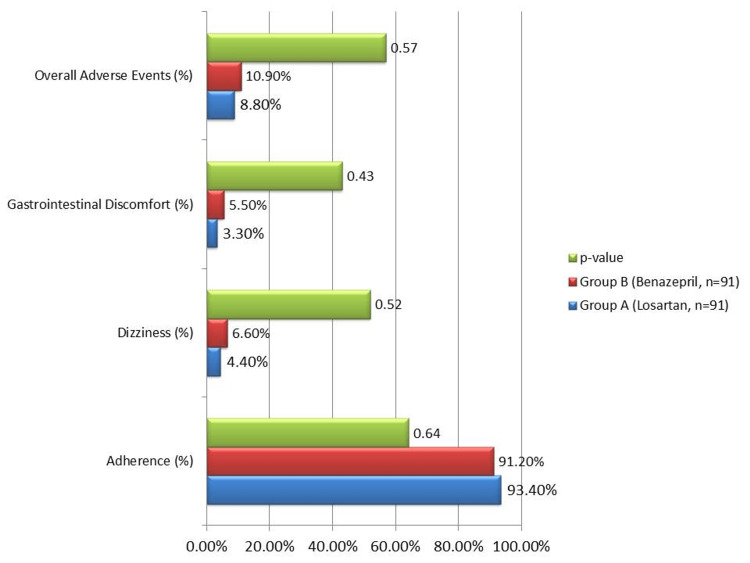
Adherence and adverse event

Adherence to treatment was high in both groups, with 85 participants (93.4%) in Group A and 83 participants (91.2%) in Group B completing the 12-month study. Adherence was assessed through patient self-reports, pill counts during monthly follow-ups, and pharmacy refill records, ensuring a comprehensive evaluation. Adherence rates did not differ significantly between groups (p = 0.64). The safety profile of both drugs is illustrated in Figure 1. The most common adverse events were dizziness and mild gastrointestinal discomfort, reported by seven participants (8.8%) in Group A and nine participants (10.9%) in Group B. However, these differences were not statistically significant (p = 0.57). All reported adverse events were mild and self-limiting, with no instances of treatment discontinuation or severe adverse events observed during the study period.

Subgroup analysis (Table 5) based on baseline BMI categories (Group A: <30 kg/m² vs. Group B: ≥30 kg/m²) revealed that participants with BMI ≥30 kg/m² exhibited smaller reductions in SBP, DBP, and HOMA-IR scores compared to those with lower BMI. Notably, the minimum SBP reduction in the higher BMI group was 21.3 mmHg, while the minimum DBP reduction was 12.4 mmHg, indicating a less pronounced treatment response. Similarly, HOMA-IR reduction was lower in obese individuals, suggesting that obesity-related factors, such as altered pharmacokinetics and chronic inflammation, may contribute to this diminished effect. These findings highlight the need for personalized therapeutic strategies to optimize outcomes in hypertensive patients with IR.

**Table 5:** Subgroup analysis by BMI Group A represents BMI <30 kg/m² and Group B represents BMI ≥30 kg/m². DBP, diastolic blood pressure; HOMA-IR, Homeostatic Model Assessment for Insulin Resistance; SBP, systolic blood pressure

Parameter	Group A	Group B	t-value	p-value	Effect size (Cohen’s d)
SBP reduction (mmHg)	29.1 ± 7.8	24.3 ± 8.2	4.12	0.000058	0.52
DBP reduction (mmHg)	17.2 ± 4.9	14.8 ± 5.1	3.85	0.000164	0.46
HOMA-IR reduction	2.1 ± 0.8	1.7 ± 0.9	3.22	0.00152	0.41

## Discussion

The findings of this study demonstrate that in hypertensive individuals with IR, LP is superior to benazepril in lowering both systolic and diastolic blood pressure and improving insulin sensitivity, as indicated by the significant reduction in HOMA-IR scores. Both medications led to an increase in HDL cholesterol (favorable lipids) without significantly raising LDL or triglycerides, supporting their metabolic safety profiles. However, losartan’s metabolic benefits were consistently greater, reinforcing its dual role in managing hypertension and metabolic disorders linked to IR.

Effect sizes for key outcomes further support the clinical relevance of these differences, with a moderate effect for SBP reduction (d = 0.52), small-to-moderate for DBP reduction (d = 0.46), and small for HOMA-IR reduction (d = 0.41). These findings highlight the therapeutic potential of ARBs in improving both cardiovascular and metabolic outcomes in hypertensive individuals with IR. While both medications exhibited good safety profiles and high adherence rates, subgroup analysis revealed that individuals with higher baseline BMI (≥30 kg/m²) experienced a less pronounced reduction in blood pressure and HOMA-IR scores, suggesting that overweight or obese patients may require personalized therapeutic approaches.

Potential alternatives, such as SGLT2 inhibitors and GLP-1 receptor agonists, have shown promise in improving cardiometabolic outcomes in individuals with IR and could be considered for combination therapy or as alternatives in specific patient populations. SGLT2 inhibitors lower blood glucose levels through renal glucose excretion [13], while GLP-1 receptor agonists enhance insulin secretion and promote weight loss [14]. These agents have demonstrated cardioprotective and nephroprotective effects, making them attractive options for patients with coexisting hypertension, obesity, and IR. However, their integration into hypertension management requires further research to assess their comparative efficacy with ARBs and ACE inhibitors.

Future studies should investigate whether combination therapy involving losartan and these novel agents may offer synergistic benefits, particularly in patients with obesity-driven metabolic dysfunction. Given the growing burden of metabolic syndrome, a shift toward comprehensive cardiometabolic treatment strategies could enhance long-term patient outcomes.

When compared with previous research, these findings support the superior efficacy of ARBs, particularly losartan, in both blood pressure reduction and metabolic improvements [15]. Prior studies suggest that ARBs may enhance insulin sensitivity by modulating insulin signaling pathways and reducing oxidative stress [16]. Although the precise molecular mechanisms remain partially understood, existing evidence implicates pathways such as PI3K/Akt and AMPK activation in the metabolic benefits of ARBs. These pathways are critical for glucose uptake and insulin-mediated cellular responses, and their dysregulation is associated with IR and metabolic syndrome.

While the current study did not directly investigate molecular mechanisms, the observed improvements in HOMA-IR scores align with the broader literature on ARBs’ potential insulin-sensitizing effects. Given the variability in mechanistic evidence across studies, further research is needed to determine the specific signaling cascades through which losartan exerts its metabolic benefits.

The observed metabolic improvements align with the renoprotective and cardioprotective effects of losartan, as noted in prior clinical trials [17]. The ability of ARBs to reduce endothelial dysfunction, mitigate inflammation, and decrease oxidative stress contributes to their broad vascular and metabolic advantages. Conversely, while ACE inhibitors like benazepril effectively lower blood pressure, their impact on insulin sensitivity and metabolic dysfunction appears less pronounced, reinforcing the therapeutic distinction between these drug classes.

This difference may be attributed to varying effects on angiotensin II signaling, which plays a dual role in vascular tone and metabolic regulation. Unlike ARBs, ACE inhibitors do not selectively block AT1 receptors, which may explain their lesser impact on IR. Given these differences, clinicians should consider individual patient metabolic profiles when selecting antihypertensive therapies, particularly for patients at high risk of diabetes and metabolic syndrome.

This study significantly contributes to the literature by emphasizing BMI as a key modifier of treatment response. While prior research has reported that obesity attenuates the antihypertensive effects of ARBs and ACE inhibitors [18], our study provides quantitative insights into BMI-related differences in both blood pressure reduction and metabolic outcomes. These findings are especially relevant in populations with high obesity and metabolic syndrome prevalence, where treatment strategies must account for individual metabolic profiles.

Previous studies indicate that both ACE inhibitors and ARBs may reduce the progression to type 2 diabetes in hypertensive patients; our study provides direct comparative evidence on their relative efficacy in improving IR - a key risk factor for diabetes [19]. Based on these results, losartan may offer a preferable therapeutic option for hypertensive individuals with early metabolic abnormalities, particularly those at high risk of developing diabetes. Future studies should explore longer-term outcomes, including the impact of losartan on diabetes prevention, cardiovascular events, and renal function in patients with hypertension and IR.

Strengths, limitations, and future directions

Strengths of the Study

This study provides valuable insights into the differential effects of LP and benazepril on blood pressure regulation and metabolic outcomes in insulin-resistant hypertensive individuals. A key strength is the direct head-to-head comparison of an ARB and an ACEI, allowing for a clear evaluation of their metabolic impacts. The study's 12-month duration provides mid-term efficacy and safety data, while the high adherence rates (over 90%) ensure that treatment effects were accurately assessed without significant confounding due to noncompliance.

The subgroup analysis based on BMI categories (<30 kg/m² vs. ≥30 kg/m²) adds a personalized medicine perspective, revealing that obese patients exhibit a less pronounced response to treatment. This highlights the need for tailored antihypertensive strategies in populations with metabolic syndrome. The use of effect size calculations strengthens the interpretation of the findings by distinguishing statistically significant changes from clinically meaningful differences. Finally, the study's focus on IR (via HOMA-IR) alongside blood pressure control aligns with the increasing emphasis on comprehensive cardiometabolic risk management in hypertensive individuals.

Limitations and Future Directions

Despite these strengths, this study has several limitations. The 12-month follow-up period, while informative, may not fully capture the long-term metabolic and cardiovascular benefits of these therapies. Existing research suggests that losartan maintains its insulin-sensitizing effects over extended periods, likely due to its role in PPAR-γ activation and oxidative stress reduction. Conversely, benazepril’s metabolic effects may diminish over time, as ACE inhibitors do not directly modulate insulin signaling pathways. Future longitudinal studies should assess whether these metabolic benefits persist, diminish, or translate into reductions in cardiovascular risk beyond the first year of treatment.

Another limitation is that only LP and benazepril were evaluated. Previous studies suggest that other ARBs (e.g., telmisartan and valsartan) and ACE inhibitors (e.g., enalapril and ramipril) exhibit distinct metabolic effects. Telmisartan, in particular, acts as a partial PPAR-γ agonist, potentially offering greater insulin-sensitizing benefits compared to other ARBs. Similarly, enalapril may provide superior glucose control among ACE inhibitors. Future studies should incorporate a broader range of these drug classes to determine whether such differences are clinically significant in insulin-resistant hypertensive patients.

This study did not assess renal function and cardiovascular outcomes, which are critical determinants of long-term treatment success. Prior research suggests that losartan may offer greater renoprotective benefits, as it reduces proteinuria and improves glomerular hemodynamics, particularly in diabetic and insulin-resistant patients. In contrast, benazepril is associated with more significant reductions in albuminuria but may not offer the same degree of cardiovascular risk reduction as ARBs. Future research should incorporate renal biomarkers, cardiovascular event tracking, and diabetes onset to comprehensively evaluate the long-term impact of these drugs.

The suggestion to investigate molecular mechanisms underlying these drug effects is particularly relevant. While this study did not directly assess intracellular signaling pathways, future research could use proteomics, transcriptomics, and biomarker analysis to explore how ARBs and ACE inhibitors differentially influence insulin-related pathways such as PI3K/Akt, AMPK activation, and oxidative stress modulation. Additionally, genetic studies could identify polymorphisms in the RAS genes that affect individual responses to these medications, enabling more personalized treatment approaches.

Given the attenuated response in overweight and obese individuals, future research should explore personalized treatment strategies for hypertensive patients with metabolic syndrome. The identification of predictive biomarkers could help optimize antihypertensive therapy, ensuring that patients receive the most effective treatment based on their metabolic profile. Furthermore, combination therapies with SGLT2 inhibitors or GLP-1 receptor agonists could provide additional benefits in managing hypertension, IR, and obesity. A multidisciplinary approach involving cardiologists, endocrinologists, and nephrologists may be key to improving outcomes in high-risk patient populations.

## Conclusions

In hypertensive individuals with IR, LP exhibited superior antihypertensive and metabolic benefits compared to benazepril, achieving greater reductions in blood pressure and HOMA-IR scores. Both agents effectively improved glycemic control and lipid metabolism; however, losartan provided enhanced insulin sensitivity without significant differences in adherence or adverse events. These findings underscore losartan’s dual therapeutic role in managing hypertension and metabolic dysfunction, supporting its preferential use in insulin-resistant hypertensive patients, particularly those at elevated risk for type 2 diabetes progression. The attenuated response observed in individuals with higher BMI highlights the potential impact of obesity on treatment efficacy, emphasizing the need for personalized therapeutic strategies. Future research should focus on long-term cardiovascular outcomes, renal function preservation, and diabetes prevention strategies to establish losartan’s potential protective effects beyond blood pressure control. Comparative studies with other ARBs (e.g., telmisartan, valsartan) and ACE inhibitors (e.g., enalapril, ramipril) are needed to determine optimal treatment selection. Additionally, given the attenuated therapeutic benefit in overweight and obese individuals, further investigation into personalized antihypertensive and metabolic management strategies is warranted. By emphasizing the interplay between hypertension, IR, and metabolic health, this study provides clinically relevant insights, reinforcing losartan’s role as a preferred therapeutic option in this high-risk population.
